# Identity centrality as a double-edged sword: mental health mechanisms among lesbian/gay and bisexual university students in China

**DOI:** 10.3389/fpsyg.2026.1737042

**Published:** 2026-06-16

**Authors:** Shanshan Zhang, Xintong Yao, Kyle Tan, Chongzheng Wei

**Affiliations:** 1 Marxism School, Sichuan University, Chengdu, China; 2 School of Psychological and Social Sciences, University of Waikato, Hamilton, New Zealand; 3 Faculty of Māori and Indigenous Studies, University of Waikato, Hamilton, New Zealand; 4 Department of Counseling Psychology, Santa Clara University, Santa Clara, CA, United States

**Keywords:** bisexuality, depressive symptoms, identity uncertainty, interpersonal discrimination, sexual identity centrality, LGB, mental health

## Abstract

**Introduction:**

This study investigated the mechanisms underlying mental health risks among lesbian/gay (LG) and bisexual university students in China, focusing on the roles of sexual identity centrality, identity uncertainty, and interpersonal discrimination in depressive symptoms.

**Methods:**

A cross-sectional survey was conducted with 363 university students (142 LG students and 221 bisexual). Group differences were examined, and structural equation modeling was used to test the direct and indirect associations among sexual orientation, sexual identity centrality, identity uncertainty, interpersonal discrimination, and depressive symptoms.

**Results:**

Compared with LG participants, bisexual participants reported lower identity centrality, higher identity uncertainty, and fewer experiences of interpersonal discrimination, whereas no significant group difference was found in depressive symptoms. Structural equation modeling indicated that identity uncertainty and interpersonal discrimination jointly mediated the association between bisexuality and depressive symptoms, with the two indirect effects operating in opposite directions and forming a competing mediation effect. Sexual identity centrality showed a double-edged role: it was associated with lower identity uncertainty but greater interpersonal discrimination, which in turn were differentially associated with depressive symptoms.

**Discussion:**

These findings reveal complex and divergent mechanisms linking sexual orientation and mental health among Chinese sexual minority university students. They also suggest that clinical interventions and future research should attend to both intrapersonal identity-related processes and interpersonal minority stress experiences when addressing depressive symptoms among LGB young adults.

## 1 Introduction

Studies on the mental health of sexual minority groups have increasingly focused on the unique psychological experiences of bisexual individuals (referred to as “Bi”) compared to lesbian and gay individuals (referred to as “LG” hereafter). Existing research has indicated that bisexual individuals outnumber lesbian women and gay men combined (Jones, 2025), and face higher psychological health risks (Feinstein and Dyar, 2017; Liow et al., 2025; Ross et al., 2018; Xu et al., 2023), including higher psychological distress (Chan et al., 2020; Taylor et al., 2019), self-injury and suicidal ideation (Xu et al., 2023), and substance abuse problems (Mongelli et al., 2019). These elevated risks are partially attributable to minority stressors specific to bisexual identity, including both interpersonal and intrapersonal experiences related below.

### 1.1 Interpersonal minority stress

The Minority Stress Model (Brooks, 1981; Frost and Meyer, 2023; Meyer, 2003) has been widely used to explain the mechanisms through which minority identity stress affects the mental health of sexual minorities. Interpersonal discrimination is recognized as a distal stressor that contributes to mental health risks for LGB groups (Dyar et al., 2019; He et al., 2023; Lattanner et al., 2022). A body of research has indicated that bisexual individuals face additional social stigma compared to their lesbian/gay counterparts (Feinstein and Dyar, 2017). Living in a monosexist society that limits a wide spectrum of sexual orientation diversity to binary options (i.e., either heterosexual or gay/lesbian), bisexual individuals are frequently denied the legitimacy and existence of their sexual orientation. As a result, they not only experience homophobia but also suffer from biphobia—such as accusations of having an unstable or inauthentic sexual orientation (Wei and Israel, 2023), or being blamed for promiscuity, irresponsibility, and indecisiveness (Israel and Mohr, 2004; Welzer-Lang, 2008). Since bisexual individuals experience interpersonal discrimination from both heterosexual and the LG communities (Arriaga and Parent, 2019), they may suffer from greater interpersonal discrimination than LG individuals (Katz-Wise and Hyde, 2012), which contributes to their negative mental health outcomes (Brewster et al., 2013; Dyar et al., 2019).

### 1.2 Sexual minority identity characteristics

In the Minority Stress Model, Meyer (2003, p. 677) emphasized that “characteristics of identity may be related to mental health both directly and in interaction with stressors.” Sexual minority identity is characterized by multidimensionality and a developmental process (Ashmore et al., 2004), affecting how individuals experience and cope with minority stress. In accounting for LGB mental health risk disparities, recent research has examined the roles of identity centrality and identity uncertainty as key psychological mechanisms influencing sexual minority mental health outcomes (Chan et al., 2020; Dyar et al., 2015; Dyar et al., 2017; Hinton et al., 2022).

Identity centrality refers to the degree to which sexual identity is important or prominent within an individual’s overall self-concept. It represents the prioritization of one’s sexual identity within one’s broader social identity hierarchies (Ashmore et al., 2004; Galupo et al., 2015). Meyer (2003) conceptualized that identity centrality may moderate the minority stress processes, such that stressors threatening a highly central LGB identity are likely to produce stronger emotional consequences (Thoits, 1999). However, empirical findings have not consistently supported this moderating hypothesis. For example, Lattanner et al. (2022) found that identity centrality did not moderate the association between interpersonal discrimination and mental health among LGB individuals. Instead, its influence on mental health operates indirectly through other mediating mechanisms (Hinton et al., 2022), producing a “double-edged sword” pattern. On one hand, higher centrality is associated with greater perceived discrimination, which in turn predicts poorer mental health outcomes (Begeny and Huo, 2017). On the other hand, higher centrality is linked to identity affirmation and greater positive affect, partly through increased outness and community engagement (Kim and Allen, 2023). Importantly, meta-analytic evidence indicates that identity centrality shows no consistent direct association with mental health outcomes, but is reliably associated with greater exposure to interpersonal discrimination and lower identity uncertainty (Hinton et al., 2022). This pattern suggests that identity centrality may shape mental health through its influence on both interpersonal (stigma exposure) and intrapersonal (identity uncertainty) processes.

Identity uncertainty refers to the extent to which an individual feels unsure about which sexual identity label best represents their patterns of sexual attraction and behaviors (e.g., gay, bisexual, or other; Dyar et al., 2017). Previous studies indicate that bisexual individuals experience greater uncertainty of their sexual identity (Chan et al., 2020; Dyar et al., 2017; Xu et al., 2021) and lower levels of identity centrality (Pew Research Center, 2013; Dyar et al., 2015) when compared to lesbian/gay people. Unlike identity centrality, identity uncertainty has been consistently identified as a proximal predictor of depressive symptoms among LGB individuals (Chan et al., 2020; Shi et al., 2022), suggesting a more direct association with psychological distress.

Furthermore, identity uncertainty (or confusion) has been identified as a normative experience during the development of a marginalized sexual identity (Cass, 1979; Troiden, 1989). According to sexual identity development theory (Troiden, 1989), identity uncertainty tends to decline as sexual orientation becomes more integrated into one’s self-concept. In combination with the robust negative correlation between identity centrality and uncertainty (Hinton et al., 2022), it is theoretically plausible that identity centrality may be related to reduced mental health risks indirectly through its association with lower levels of identity uncertainty.

Taken together, existing literature suggests bisexual individuals report higher levels of interpersonal discrimination (Katz-Wise and Hyde, 2012) and identity uncertainty (Chan et al., 2020) but lower identity centrality relative to lesbian and gay individuals (Pew Research Center, 2013; Dyar et al., 2015). In the present study, we conceptualized identity centrality not as an independent predictor of depression, but as an important identity characteristic that influences minority stress processes. Lower identity centrality may affect mental health risk for bisexual individuals via its concurrent associations with heightened identity uncertainty and less interpersonal discrimination. This framework informs the present study and highlights the need to test these simultaneous pathways.

### 1.3 Gender differences within LGB groups

Within the cisgender LGB community, women generally experience greater social acceptance (Friedman et al., 2014; Monto and Supinski, 2014), have a greater willingness to come out (Arena and Jones, 2017), and show lower levels of social discrimination and internalized negativity than sexual minority men (Baams et al., 2015; Paul et al., 2014; Xu et al., 2022). Conversely, prior research revealed that sexual minority women tend to experience equal or higher mental health risks compared to sexual minority men (Chan and Leung, 2023; Chan et al., 2020; Lucassen et al., 2017; Lu et al., 2023; Shi et al., 2022), which raises the question: why do sexual minority women exhibit heightened vulnerability to mental health when they experience lower levels of social stigma than men?

One possible explanation is the sensitivity of the interplay of gender and sexual orientation on minority stress. For instance, Shi et al. (2022) examined a large sample of Chinese university students and found that the degree of correlation between LGB sexual orientation and depressive symptoms was stronger for women than men; conversely, among peers who were unsure of their sexual orientation, men reported a higher risk than women. Recently, research has shifted from analyzing group-level gender differences to exploring the intersectionality of gender and sexual orientation on the minority stressors (Pu et al., 2025b; Xu et al., 2022). This approach helps further elucidate the heterogeneity of mental health risk mechanisms within the LGB population.

### 1.4 The Chinese cultural context

In Mainland China, the absence of government recognition for sexual and gender minority rights, such as marriage equality and anti-discrimination policies, results in relatively lower acceptance of LGBTQ+ individuals (Flores, 2021). While the Chinese government maintains restrictive measures on LGBT+ topics in public discourse and school-based sex education (Cui, 2023; Qin and Zhang, 2023), queer media culture has created spaces for non-normative sexual and gender narratives (Zhao, 2020), which have partially fostered greater public awareness and tolerance of LGBT+ communities in China (Lin et al., 2016). In particular, being female, young, and having a higher level of education are significant predictors of greater support for LGBT+ rights (Meyer et al., 2024). This trend may also be attributed to the fact that, unlike in Western cultures, the stigmatization of sexual minorities in China does not stem from religious taboos, but arises mainly from the conflict with the Confucian values centered on carrying the family line and heteronormative marriage norms (Lin et al., 2016). Therefore, the social stigma felt by Chinese sexual minorities may vary depending on their interpersonal environments shaped by both the salience of gender roles in family responsibilities and the timing of life events (such as entering college or reaching marriageable age).

Recently, a series of studies have explored the intragroup disparities on socio-cultural stigma stress between the Chinese bisexuals and lesbian/gay individuals, while also highlighting the role of gender. Since men are viewed as the primary bearers of the family lineage in Confucian cultures, which confines male sexuality to heteronormative norms more strictly than female sexuality, Chinese gay and bisexual men generally face harsher individual stigma and interpersonal discrimination than sexual minority women (Chan and Huang, 2022; Xu et al., 2022). Chinese bisexual individuals were confirmed to face dual discrimination from both monosexism and heterosexism (Chan and Leung, 2023). However, there is no direct evidence demonstrating that Chinese bisexual individuals experience greater interpersonal discrimination than their LG counterparts (Pu and Xu, 2024), nor has any study established a link between such discrimination disparities and their mental health outcomes.

Meanwhile, the sexual identity characteristics have received relatively less attention yet have been evidenced to contribute to the mental health disparities among Chinese LGB groups (i.e., identity uncertainty and centrality; Chan et al., 2020; Pu et al., 2025a). Unlike the extensive attention given to identity centrality in international research (Hinton et al., 2022), no study has yet examined its mechanisms linking external discrimination and internal identification factors in the Chinese context, which may contribute to designing more effective mental health interventions for Chinese LGB people.

### 1.5 The present study

Although interpersonal discrimination and identity-related factors have been identified as key contributors to mental health outcomes among LGB populations (Chan et al., 2020; Hinton et al., 2022; Lattanner et al., 2022; Meyer, 2003; Wei and Israel, 2023), few research to date has examined how sexual identity centrality, identity uncertainty, and experiences of interpersonal discrimination interact to shape mental health disparities between lesbian/gay and bisexual individuals.

Responding to calls to extend the Minority Stress Model by integrating psychological mediation processes that elucidate how minority stigma “gets under the skin” (Frost and Meyer, 2023; Hatzenbuehler, 2009), and drawing on sexual identity development frameworks that emphasize the dynamic interplay among identity dimensions (Troiden, 1989), the present study articulates a conceptual ordering by positioning identity centrality as an upstream factor that influences subsequent intrapersonal (identity uncertainty) and interpersonal (discrimination) stress processes. Specifically, this study aimed to test three theory-driven expectations: (1) there would be differences between cisgender bisexual individuals and lesbian/gay individuals in mainland China in characteristics of minority identity (i.e., uncertainty and centrality), interpersonal discrimination, and depressive symptoms; (2) sexual orientation would be linked to depressive symptoms through sexual identity uncertainty and interpersonal discrimination; and (3) Identity centrality would be indirectly linked to depressive symptoms through identity uncertainty and interpersonal discrimination. In addition, given the limited prior research on the joint role of sexual orientation and gender in psychological risk mechanisms, we conducted exploratory analyses to examine potential gender differences in these pathways.

## 2 Methods

### 2.1 Procedure

This study was part of a larger project to understand Chinese university students’ attitudes and psychological development so as to improve and refine the design and pedagogy of undergraduate sexuality education curricula. This study received ethical approval from the Medical Ethics Committee of [MASKED FOR REVIEW] University. We disseminated an online questionnaire via Wenjuanxing, a widely used survey platform. Students were recruited at a comprehensive university in southwestern region mainland China via students’ course group chats and the researchers’ professional networks. Participation in the questionnaire was voluntary and anonymous. Participants provided informed consent electronically before beginning the survey. Upon completion, all participants received a 2 RMB cash reward via Wenjuanxing.

### 2.2 Participants

A total of 1,957 questionnaires were collected from May to July 2024. Screening was conducted to ensure data quality. Responses were excluded if they failed the attention check questions (e.g., reporting the number of digits in their student ID number, *N* = 78), chose the same response option for all the items of a scale (*N* = 65), and completed the survey within an unreasonably short amount of time (*N* = 1). The present study focused on cisgender lesbian, gay, and bisexual students. Therefore, heterosexual respondents (*N* = 1,388) were excluded from the analytic sample. In addition, 62 participants were removed as they did not meet our inclusion criteria, including respondents who identified as transgender (*n* = 20), asexual (*n* = 10), questioning (*n* = 8), pansexual (*n* = 2), non-binary (*n* = 1), and 21 participants who selected “other” but did not provide sufficient information about their sexual orientations and/or gender identities for recoding.

The final valid sample consisted of 49 lesbian women, 93 gay men, and 221 bisexual individuals including 175 bisexual women and 46 bisexual men. There was a significant difference in gender distribution between Bi and L/G participants (*χ*^2^ = 73.03, *p* < 0.001). Specifically, there was a higher proportion of bisexual women compared to bisexual men, and more gay men than lesbian women. No other significant differences in demographic characteristics. For more information, please see Table 1.

**Table 1:** Demographics of the participants.

Variable	Total (*n* = 363)	Bi (*n* = 221)	LG (*n* = 142)	Group difference *χ*^2^/*t*-value
	*N* (%)/Mean (S.D.)	*N* (%)/Mean (S.D.)	*N* (%)/Mean (S.D.)	
Gender, *n* (%)				73.03^***^
Women	224 (61.71)	175 (79.19)	49 (34.51)	
Men	139 (38.29)	46 (20.81)	93 (65.49)	
Education level				0.13
Postgraduates	75 (20.66)	47 (21.27)	28 (19.72)	
Undergraduates	288 (79.34)	174 (78.73)	114 (80.28)	
Major				3.45
Humanities	118 (32.5%)	70 (31.67%)	48 (33.8%)	
Science and Engineering	157 (43.3%)	91 (41.18%)	66 (46.48%)	
Medicine	60 (16.5%)	39 (17.65%)	21 (14.79%)	
Arts and Sports	28 (7.7%)	21 (9.5%)	7 (4.93%)	
Place of origin				0.44
Rural	86 (23.7%)	51 (23.08%)	35 (24.65%)	
Township	80 (22%)	47 (21.27%)	33 (23.24%)	
Urban	197 (54.3%)	123 (55.66%)	74 (52.11%)	
Parents’ education level				5.35
Both under high school education	72 (19.8%)	42 (19%)	30 (21.13%)	
One has high school education	89 (24.5%)	47 (21.27%)	42 (29.58%)	
One has college diploma or above	60 (16.5%)	36 (16.29%)	24 (16.9%)	
Both have college diploma or above	142 (39.1%)	96 (43.44%)	46 (32.39%)	
Centrality (1–6)	3.21 ± 1.19	2.76 ± 1.03	3.89 ± 1.09	9.93^***^
Uncertainty (1–6)	2.51 ± 1.33	2.92 ± 1.28	1.87 ± 1.14	−8.20^***^
Interpersonal discrimination (1–6)	1.72 ± 0.87	1.44 ± 0.66	2.15 ± 0.98	7.53^***^
Depressive symptoms (1–4)	2.10 ± 0.50	2.08 ± 0.50	2.13 ± 0.51	0.84

LG, Lesbian Women and Gay men; Bi, Bisexual individuals.

^*^p < 0.05, ^**^p < 0.01, ^***^p < 0.001.

### 2.3 Measurements

Sexual and gender identity were assessed using two questions adapted to the Chinese sociolinguistic context. Participants’ assigned sex at birth was assessed by using one question, “What’s your biological sex?” with response options being male and female, as Chinese birth certificates only have these two options. We used the term “biological sex” rather than “sex assigned at birth” because it is more familiar to Chinese young people and is more consistent with the terminology commonly used in official registration documents. Participants were then asked to indicate their sexual identity (“性身份”) by selecting from the following options: heterosexual, lesbian/gay, bisexual, transgender, or other, with a write-in option. Multiple selections were allowed. In everyday Chinese usage, the term “性身份” is often used broadly to refer to both sexual orientation and gender-related identities. These two items were used to screen for cisgender LGB participants. However, in this sample, no participants selected both a transgender identity and an LGB identity. Prior to formal data collection, we piloted the survey with both heterosexual and sexual minority university students, who reported that the questions were clear and understandable.

Depressive symptoms were assessed by the Center for Epidemiologic Studies Depression Scale (CES-D, 10-item version; Kohout et al., 1993), with an example item being “I felt sad.” This scale demonstrates strong structural validity among Chinese university students (Yu et al., 2013) and has been employed in studies measuring depressive symptoms in non-clinical sexual minority populations (Paul et al., 2014; Shramko et al., 2018). Items were rated on a 4-point Likert scale (1 = “not at all”, 4 = “almost always”), with higher scores indicating more severe depression. In the present study, the scale demonstrated good internal consistency, with a Cronbach’s alpha of 0.87.

Identity centrality was assessed using the Identity Centrality Subscale of the Lesbian, Gay, and Bisexual Identity Scale (LGBIS) (Mohr and Kendra, 2011), which assesses the importance of sexual identity within one’s self-concept. It includes five items (e.g., “My sexual orientation is a central part of my identity”) and uses a 6-point rating scale (1 = “strongly disagree”, 6 = “strongly agree”). The Cronbach’s alphas of this scale ranged from 0.83 to 0.86 in the original study (Mohr and Kendra, 2011), and it was 0.80 in the current study.

Identity uncertainty was assessed by the Identity Uncertainty Subscale of the Lesbian, Gay, and Bisexual Identity Scale (LGBIS) (Mohr and Kendra, 2011), which included four items (e.g., “I feel very confused when trying to figure out my sexual orientation”) and uses a 6-point rating scale (1 = “strongly disagree”, 6 = “strongly agree”), with higher scores indicating greater difficulty in defining one’s sexual identity. The Cronbach’s alpha in the original study ranged from 0.88 to 0.93 (Mohr and Kendra, 2011) and was 0.91 in the present study.

Interpersonal discrimination experiences were assessed by the Hostility subscale of the Brief Anti-Bisexual Experiences Scale (Brewster and Moradi, 2010; Dyar et al., 2019). This scale was originally designed to assess interpersonal discrimination experienced by bisexual individuals and demonstrated good reliability (Dyar et al., 2019). In this study, we adapted its items to apply to both LG and Bi individuals (e.g., “Others have acted uncomfortable around me because of my bisexuality/homosexuality”). The three items used a 6-point rating scale (1 = “never”, 6 = “almost all the time”), with higher scores indicating more experiences of interpersonal discrimination. Measurement invariance testing supported a unidimensional structure and demonstrated configural and metric invariance of the Hostility subscale across the Bi and LG groups, indicating that the scale functioned comparably across groups (see Supplementary Table S1). This subscale also demonstrated high internal reliability in this study (Cronbach’s alpha = 0.91).

### 2.4 Common method bias test

Since all variables in this study were measured using self-report questionnaires, we conducted Harman’s single-factor test to examine the potential influence of common method bias (CMB) (Podsakoff et al., 2003). An exploratory factor analysis (EFA) was performed on all the measurement items, and the first factor accounted for only 23.58% of the total variance, which is below the commonly recommended threshold of 50%. Therefore, common method bias is unlikely to be a serious concern in this study.

### 2.5 Data analysis

Descriptive statistics were used to characterize the demographics and measured variables for the analyzed sample. Chi-square tests were used to compare lesbian/gay and bisexual individuals on demographics. Independent-samples *t* tests and one-way ANOVAs were conducted to examine differences in the outcome variables by demographic characteristics (see Tables 1, 2 and Supplementary Tables S2–S5). Spearman’s zero-order correlations were computed to examine associations among the study variables (see Table 3). Detailed codes for analyses related to demographic variables are presented in the table note.

**Table 2:** Gender differences in key study variables.

Variable	Total				LG				Bi			
	Men *M* ± SD	Women *M* ± SD	*t* (df)	*p*	Men *M* ± SD	Women *M* ± SD	*t* (df)	*p*	Men *M* ± SD	Women *M* ± SD	*t* (df)	*p*
Depressive symptoms	2.08 ± 0.54	2.11 ± 0.48	−0.53 (361)	0.595	2.14 ± 0.52	2.11 ± 0.50	0.35 (140)	0.73	1.97 ± 0.55	2.11 ± 0.48	−1.76 (219)	0.081
Identity uncertainty	2.10 ± 1.21	2.77 ± 1.34	−4.81 (361)	**< 0.001**	1.73 ± 1.02	2.14 ± 1.32	−2.06 (140)	**0.041**	2.85 ± 1.24	2.94 ± 1.29	−0.46 (219)	0.65
Identity centrality	3.50 ± 1.14	3.03 ± 1.18	3.74 (361)	**< 0.001**	3.85 ± 1.04	3.98 ± 1.19	−0.70 (140)	0.487	2.79 ± 1.02	2.76 ± 1.04	0.20 (219)	0.845
Interpersonal discrimination	2.06 ± 1.01	1.50 ± 0.69	5.74 (218.13)	**< 0.001**	2.33 ± 1.01	1.80 ± 0.81	3.20 (140)	**0.002**	1.52 ± 0.77	1.42 ± 0.63	0.91 (219)	0.367

Significant *p* values are bolded.

**Table 3:** Intercorrelations among variables.

Variables	1	2	3	4	5	6	7	α
1. Uncertainty	—							0.91
2. Centrality	−0.318**	—						0.8
3. Interpersonal discrimination	−0.153**	0.305**	—					0.91
4. Depressive symptoms	0.129*	0.042	0.261**	—				0.87
5. Gender	0.245**	−0.193**	−0.313**	0.028	—			
6. Sexual orientation	0.388**	−0.463**	−0.395**	−0.044	0.449**	—		
7. Education level	0.048	0.007	−0.088	−0.032	0.052	0.019	—	
8. Parents’ education level	−0.046	−0.059	−0.016	−0.041	0.127*	0.099	−0.182**	

Binary variables were coded as follows: Sexual orientation (LG = 0, Bi = 1), Gender (men = 0, women = 1), and Education level (undergraduate = 0, postgraduate = 1). Parents’ education level was recoded into a four-point ordered variable and treated as a continuous variable in the analyses, with higher scores indicating higher levels of parental educational attainment. Because Major and Place of origin were multicategory categorical variables, their associations with the focal study variables were examined using one-way ANOVAs and the results were reported in the Supplementary Tables.

^*^p < 0.05, ^**^p < 0.01.

Subsequently, hierarchical multiple regression models were used to predict the depressive symptoms difference between lesbian/gay and bisexual individuals. In the first block, major and parents’ education level were entered as covariates based on preliminary analyses, which showed small but significant differences in identity centrality across major groups (see Supplementary Table S2) and a small but significant association involving parents’ education level and gender (see Table 3). Other demographic variables, such as place of origin and participants’ education level, were not significantly related to the key study variables and were therefore not included. In the second block, we examined the main effects and interaction effects of sexual orientation and gender on depressive symptoms. We then included sexual identity characteristics (i.e., identity uncertainty and centrality) and interpersonal discrimination experiences as the predictors of depressive symptoms in the third block of the analysis. In addition, sexual identity characteristics and interpersonal discrimination were regressed on sexual orientation, gender, and their interaction.

Structural equation modeling (SEM) was employed to assess the multiple mediation pathways (i.e., identity uncertainty, centrality, and interpersonal discrimination experiences) linking sexual orientation and depressive symptoms (Kline, 2023). The fit of the hypothesized model to the data was evaluated by the comparative fit index (CFI), the Tucker-Lewis index (TLI), the root mean square error of approximation (RMSEA), and the standardized root mean square residual (SRMR) (West et al., 2012). The bootstrapping analysis was conducted using 5,000 bootstrap samples to estimate the indirect effects of sexual orientation on depressive symptoms via the three variables. Bias-corrected 95% confidence intervals (CIs) of the parameter estimates were used to determine the significance of the indirect effects (Cheung and Lau, 2007).

To facilitate cross-scale comparisons, all variables were represented by mean item scores in the analyses, except in the SEM, where latent variables were constructed from the original item scores. Data analyses were performed using SPSS 25.0 and M*plus* 8.3. The codes used for data analysis are available in the Supplement.

## 3 Results

### 3.1 Sexual orientation and gender differences on key variables

As presented in Table 1, compared with the LG group, the Bi group reported significantly lower levels of sexual identity centrality and interpersonal discrimination, but significantly higher identity uncertainty. There was no significant difference in depressive symptom levels between the LG and Bi groups.

As shown in Table 2, no significant gender difference was found in depressive symptoms among LGB participants. However, women reported higher identity uncertainty than men, whereas men reported higher identity centrality and interpersonal discrimination than women. Further within-group analyses showed that the gender difference in identity uncertainty and interpersonal discrimination was significant only within the LG group, but not within the bisexual group. Specifically, lesbian women reported lower levels of interpersonal discrimination and higher levels of identity uncertainty than gay men, whereas bisexual women and men did not significantly differ on either variable.

### 3.2 Associations of sexual orientation, gender, sexual identity characteristics, interpersonal discrimination, and depressive symptoms

Table 3 shows the intercorrelations of the measured variables. Depressive symptoms were positively correlated with sexual identity uncertainty (*r* = 0.129, *p* < 0.05) and interpersonal discrimination experiences (*r* = 0.261, *p* < 0.01) but were unrelated to identity centrality or demographic characteristics. Identity centrality was positively correlated with interpersonal discrimination (*r* = 0.305, *p* < 0.05) and negatively correlated with identity uncertainty (*r* = −0.318, *p* < 0.01). Identity uncertainty was also negatively correlated with interpersonal discrimination.

In terms of demographic variables, both gender and sexual orientation were positively associated with identity uncertainty but negatively associated with identity centrality and interpersonal discrimination. Parents’ education level showed small but significant associations with gender and education level, while participants’ education level was not significantly associated with key study variables.

Table 4 presents the results of hierarchical multiple regression models testing the associations between sexual orientation, gender, sexual identity attributes (i.e., identity uncertainty and centrality), interpersonal discrimination experiences, and depressive symptoms. In Step 1, neither major nor parents’ education level significantly predicted depressive symptoms, *R*^2^ = 0.002, *F*(4, 358) = 0.174, *p* = 0.952. In Step 2, sexual orientation and gender did not significantly predict depressive symptoms, nor did the interaction term (sexual orientation x gender). This model resulted in a small and non-significant 1% increase in the explained variance in depressive symptoms, but this increment was not significant, *ΔR*^2^ = 0.01, *ΔF*(3, 355) = 1.241, *p* = 0.295. In the final model, both sexual identity uncertainty (*β* = 0.163, *p* = 0.004) and interpersonal discrimination (*β* = 0.299, *p* < 0.001) significantly predicted increased depressive symptoms, whereas identity centrality did not significantly predict depressive symptoms (*β* = 0.01, *p* = 0.87), leading to a significant additional increase of 9.6% in explained variance, *ΔR*^2^ = 0.096, *ΔF*(3, 352) = 12.70, *p* < 0.001. The final model explained 10.9% of the total variance in depressive symptoms.

**Table 4:** Hierarchical multiple regression model results.

Variable	Depressive symptoms		Uncertainty		Centrality		Interpersonal discrimination	
	*B* (SE)	*β*	*B* (SE)	*β*	*B* (SE)	*β*	*B* (SE)	*β*
Block 1: Control variables								
Parents’ education level	−0.14 (0.23)	−0.032	−0.086 (0.056)	−0.076	−0.010 (0.048)	−0.01	0.024 (0.036)	0.032
Major (science and engineering)	−0.03 (0.61)	−0.003	0.228 (0.150)	0.085	−0.444 (0.130)	−0.185**	0.045 (0.098)	0.026
Major (medicine)	−0.14 (0.78)	−0.01	−0.077 (0.194)	−0.021	−0.312 (0.168)	−0.098	0.107 (0.127)	0.046
Major (arts and sports)	−0.21 (1.05)	−0.011	−0.599 (0.260)	−0.12*	−0.050 (0.225)	−0.011	0.218 (0.169)	0.067
Block 2								
Sexual orientation								
Bisexual (lesbian/Gay as reference)	−0.89 (0.97)	−0.086	1.142 (0.220)	0.42***	−0.963 (0.191)	−0.396***	−0.837 (0.144)	−0.471***
Gender								
Women (Men as reference)	0.44 (0.90)	0.042	0.548 (0.216)	0.201*	0.109 (0.187)	0.045	−0.569 (0.141)	−0.319***
Interaction								
Sexual orientation x Gender	1.13 (1.20)	0.112	−0.339 (0.295)	−0.128	−0.281 (0.256)	−0.118	0.471 (0.192)	0.271*
Block 3								
Uncertainty	0.62 (0.22)	0.163**						
Centrality	0.04 (0.25)	0.01						
Interpersonal discrimination	1.73 (0.33)	0.299***						

Major was dummy coded, with Humanities as the reference group. Parents’ education level was recoded into a four-point ordered variable and treated as a continuous variable in the analyses, with higher scores indicating higher levels of parental educational attainment.

^*^p < 0.05, ^**^p < 0.01, ^***^p < 0.001.

Additional analyses were conducted to examine whether sexual orientation and gender accounted for variance in characteristics of minority identity characteristics (i.e., uncertainty and centrality) and perceived interpersonal discrimination. Sexual orientation negatively predicted identity centrality and interpersonal discrimination, while positively predicted identity uncertainty, revealing that bisexual individuals reported lower levels of identity centrality (*β* = −0.396, *p* < 0.001), fewer experiences of interpersonal discrimination (*β* = −0.471, *p* < 0.001), and higher levels of identity uncertainty compared to their LG counterparts (*β* = 0.42, *p* < 0.001). The main effect of gender was positive for identity uncertainty and negative for interpersonal discrimination, indicating that LGB women reported higher levels of identity uncertainty (*β* = 0.201, *p* = 0.011) and fewer experiences of interpersonal discrimination than LGB men (*β* = −0.319, *p* < 0.001). Furthermore, no significant interaction effects between gender and sexual orientation were found for identity uncertainty or centrality, but a significant interaction emerged for interpersonal discrimination (*β* = 0.271, *p* = 0.015).

Building on the gender group comparisons reported above (see Table 2), these findings suggest that gender and sexual orientation jointly shape interpersonal discrimination, as evidenced by a significant interaction and subgroup differences confined to the LG group. In contrast, identity uncertainty appeared to be influenced by gender and sexual orientation in a more additive manner, with significant main effects but no evidence of interaction.

### 3.3 The mediation model examining differences in depressive symptoms mechanisms between the LG and Bi groups

**Figure 1:**
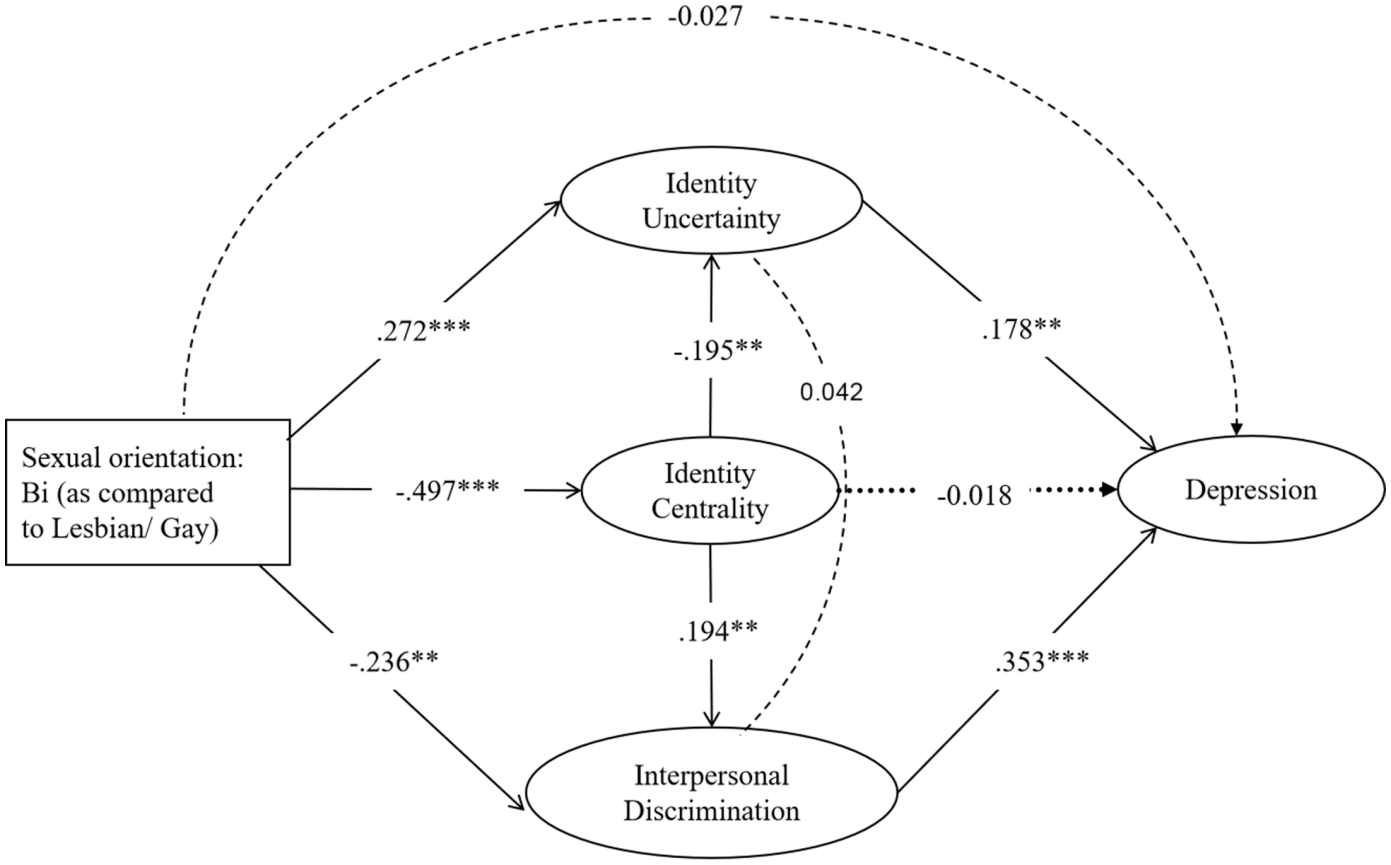
Multiple mediation SEM diagram controlling for gender. ^*^p < 0.05, ^**^p < 0.01, ^***^p < 0.001.

A multiple mediation model was developed to test the interrelationships among identity centrality, uncertainty, and interpersonal discrimination, and to explore their mediating roles in the association between sexual orientation and depressive symptoms (see Figure 1). Firstly, the measurement model of the three predictors and one dependent variable was measured. All factor loadings from the observed indicators to their respective latent variables were statistically significant (*βs* = 0.457–0.919, *p* < 0.001), and the overall model fit was satisfactory: *χ*^2^ = 530.49 (df = 203, *p* < 0.001), CFI = 0.92, TLI = 0.91, RMSEA = 0.067, and SRMR = 0.056.

In the multiple mediation model, gender was included as a covariate because it showed robust associations with key study variables and was theoretically relevant to sexual identity development and minority stress processes.^1^ The model demonstrated acceptable fit to the data, χ^2^(239) = 594.238, *p* < 0.001, CFI = 0 0.916, TLI = 0.903, SRMR = 0.056, and RMSEA = 0.064. The results showed that (see Table 5), relative to identifying as lesbian or gay, identifying as bisexual was positively associated with identity uncertainty (*β* = 0.272, *p* < 0.001) but negatively associated with identity centrality (*β* = −0.497, *p* < 0.001) and interpersonal discrimination (*β* = −0.236, *p* < 0.001). Identity centrality was negatively related to identity uncertainty (*β* = −0.195, *p* < 0.01) yet positively related to interpersonal discrimination (*β* = 0.194, *p* < 0.01). Both identity uncertainty (*β* = 0.178, *p* < 0.001) and interpersonal discrimination (*β* = 0.353, *p* < 0.001) were significantly associated with greater depressive symptoms. The direct effects of sexual orientation and identity centrality on depressive symptoms were not significant.

**Table 5:** Parameter estimates for the hypothesized model (controlling gender).

Path	*β*/estimate	S.E.	*p*/95% bootstrap CI
Direct path	*β*	S.E.	*p*
Centrality → uncertainity	−0.195**	0.074	0.008
Centrality → IPH	0.194**	0.073	0.008
Uncertainity → depressive symptoms	0.178**	0.068	0.009
Centrality → depressive symptoms	−0.018	0.075	0.814
IPH → depressive symptoms	0.353***	0.076	< 0.001
SO → uncertainity	0.272***	0.071	< 0.001
SO → centrality	−0.497***	0.056	< 0.001
SO → IPH	−0.236**	0.069	0.001
IPH ↔ uncertainity	0.042	0.076	0.583
SO → depressive symptoms	−0.027	0.072	0.705
Gender → uncertainity	0.108†	0.055	0.05
Gender → IPH	−0.187**	0.056	0.001
Gender → centrality	0.045	0.06	0.454
Gender → depressive symptoms	0.118*	0.06	0.048
Indirect path	Estimate	S.E.	95% bootstrap CI
SO → uncertainty → depressive symptoms	0.054*	0.026	[0.013, 0.116]
SO → centrality→ depressive symptoms	0.01	0.042	[−0.073, 0.094]
SO → IPH → depressive symptoms	−0.093**	0.035	[−0.174, −0.033]
Centrality → uncertainty → depressive symptoms	−0.023*	0.013	[−0.055, −0.003]
Centrality → IPH → depressive symptoms	0.046*	0.022	[0.011, 0.096]
SO→ Centrality → uncertainty → depressive symptoms	0.019*	0.011	[0.002, 0.046]
SO→ centrality → IPH → depressive symptoms	−0.038*	0.017	[−0.078, −0.010]
Total indirect effect	−0.048	0.054	[−0.152, 0.063]
Total effect	−0.079	0.071	[−0.216, 0.057]

SO, Sexual Orientation; IPH, Interpersonal discrimination. *β* values are STDYX standardized coefficients for direct structural paths. Indirect effects are reported as unstandardized estimates with bias-corrected bootstrap 95% confidence intervals (5,000 resamples).

^*^p < 0.05, ^**^p < 0.01, ^***^p < 0.001, ^†^*p* < 0.10.

Bootstrap method for testing indirect effects (Zhao et al., 2010) showed that interpersonal discrimination significantly mediated the relationship between sexual orientation and depressive symptoms, *B* = −0.093, 95% CI [−0.174, −0.033], as did sexual identity uncertainty, *B* = 0.054, 95% CI [0.013, 0.116]. Specifically, compared to lesbian and gay individuals, bisexual individuals showed lower depressive symptoms via interpersonal discrimination but higher depressive symptoms via identity uncertainty. The two indirect paths from sexual orientation to depressive symptoms present opposite directions, with no significant direct effect (*β* = −0.027, *p* = 0.705). Identity centrality also influences depressive symptoms through two mediating pathways operating in opposite directions: one via reduced identity uncertainty [*B* = −0.023, 95% CI (−0.055, −0.003)] and the other via increased interpersonal discrimination [*B* = 0.046, 95% CI (0.011, 0.096)], with no significant direct effect (*β* = −0.018, *p* = 0.814).

Furthermore, we tested two chained indirect paths from sexual orientation to depressive symptoms. The first path was sequentially mediated by sexual identity centrality and interpersonal discrimination, *B* = −0.038, 95% CI [−0.078, −0.010]. The negative indirect effect indicates that bisexual individuals experienced lower depressive symptoms via reduced identity centrality and subsequent decreases in interpersonal discrimination relative to LG individuals. The second path was mediated by identity centrality and uncertainty, *B* = 0.019, 95% CI [0.002, 0.046]. The positive indirect effect suggests a modest countervailing pathway whereby lower identity centrality was associated with elevated identity uncertainty and, in turn, greater depressive symptoms. The total structural equation model explained approximately 13.2% of the variance in depressive symptoms.

### 3.4 Ad-hoc analyses of gender differences in the mediation model

To further examine the robustness of the overall pattern and probe potential gender differences in the mediating mechanisms, ad-hoc SEM analyses were conducted separately for LGB men and women. The results (see Supplementary Tables S6–S8) showed that both gender-stratified models showed acceptable fit. The association between sexual orientation and identity centrality was consistently significant across gender groups. However, the paths from sexual orientation to identity uncertainty (*β* = 0.400, *p* < 0.001) and interpersonal discrimination (*β* = −0.332, *p* < 0.001) were significant only among men, not among women. In contrast, the paths from identity centrality to identity uncertainty (*β* = −0.244, *p* = 0.022) and interpersonal discrimination (*β* = 0.305, *p* = 0.002) were significant only among women but not among men. This pattern suggests that the multiple mediating pathways may vary by gender. In particular, identity centrality appears to function as a more consequential organizing identity characteristic among women, whereas among men, sexual orientation itself seems to be more proximally tied to minority stress processes.

To formally examine whether these effects differed by gender, a multi-group SEM was conducted in which the paths from sexual orientation and identity centrality to uncertainty and interpersonal discrimination were estimated separately for men and women. The gender-stratified analyses suggested differences in the pattern of significant pathways across men and women. However, formal multi-group SEM tests indicated that these gender differences were not statistically significant. Thus, while there is some descriptive evidence of variation in how identity processes operate across gender, the overall mediation structure does not significantly differ between men and women (see Table 6).

**Table 6:** Between-group comparisons of key structural paths across gender.

Path	Men estimate (*β*)	S.E.	*p*	Women estimate (*β*)	S.E.	*p*	Difference (men − women)	S.E.	*p*
SO → uncertainty	1.094***	0.239	0	0.61*	0.279	0.029	0.484	0.366	0.186
SO → IPH	−0.484**	0.148	0.001	−0.187	0.121	0.121	−0.297	0.183	0.106
Centrality → uncertainty	−0.205	0.153	0.18	−0.414*	0.192	0.031	0.209	0.239	0.382
Centrality → IPH	0.181†	0.1	0.069	0.167*	0.068	0.014	0.014	0.120	0.907
Uncertainty → depressive symptoms	0.035	0.037	0.336	0.056†	0.029	0.05	−0.021	0.046	0.651

SO, Sexual Orientation; IPH, Interpersonal discrimination. ^*^p < 0.05, ^**^p < 0.01, ^***^p < 0.001, ^†^*p* < 0.10.

## 4 Discussion

This study examined the mechanisms by which sexual identity uncertainty, centrality, and interpersonal discrimination influence depressive symptoms among bisexual (Bi) and lesbian and gay (LG) Chinese university students. To the best of our knowledge, this was the first study that found sexual identity centrality to be associated with both greater interpersonal discrimination experiences and lower identity uncertainty, which in turn were related to depressive symptoms among Chinese LGB young adults.

### 4.1 Overview of differences between LG and bisexual groups

Relative to identifying as LG, identifying as bisexual was positively associated with identity uncertainty, which in turn was associated with greater depressive symptoms. At the same time, bisexual identification was negatively associated with interpersonal discrimination, which in turn was associated with fewer depressive symptoms among bisexual individuals. These two indirect pathways operated in opposite directions, resulting in a statistically non-significant total effect of sexual orientation on depressive symptoms. This pattern represents a competing mediation model (Zhao et al., 2010), in which distinct minority stress mechanisms simultaneously elevate and reduce psychological risk among bisexual individuals. Importantly, this finding does not imply that bisexual individuals experience less overall minority stress. Rather, it suggests that different stress processes (internal identity-related uncertainty vs. external discrimination exposure) may operate at varying magnitudes within this population.

At first glance, no significant difference in depressive symptoms was found between the Bi and LG groups, which may be attributed to sampling bias, as a prior meta-analysis study found that the mental health disparities between Bi and LG individuals have a small effect size (Ross et al., 2018). Additionally, this difference might not be pronounced in our research sample of university students who reported low levels of depressive symptoms overall. Future research could include clinical samples and more mental health indicators, such as anxiety and life satisfaction, to examine the mental health variations between bisexual and LG groups. Rather than focusing on the overall mental health differences between the two groups, it is more important to examine how the underlying minority stress mechanisms differ between Bi and LG individuals to better understand subgroup differences and the unique challenges experienced by bisexual people.

At the interpersonal level, though the prior research did demonstrate that Chinese bisexuals are subjected to pressures stemming from both monosexism and heterosexism (Chan and Leung, 2023; Wei and Israel, 2023), young bisexual university students in our study reported significantly lower levels of interpersonal discrimination experiences than their LG counterparts. This pattern contradicted previous study results (Katz-Wise and Hyde, 2012) but aligned with findings from a national survey with an American LGB sample (Pew Research Center, 2013). Such a finding may be related to a lower level of outness among bisexual people compared to lesbian/gay individuals (Chan et al., 2020), which may reduce exposure to overt discrimination. In this sense, the lower levels of interpersonal discrimination reported by bisexual individuals may be influenced by multiple minority stress processes that were not captured in the present study.

At the intrapersonal level, our findings corroborated previous research that bisexual individuals report lower sexual identity centrality and higher uncertainty than their LG counterparts (Chan et al., 2020; Dyar et al., 2015). Sexual identity uncertainty has been identified as a high-risk factor for the mental health of bisexual university students. Such uncertainty may reflect individuals’ lack of clarity in their self-concept (Campbell et al., 1996), which can erode the emotional regulation skills necessary to cope with depressive symptoms and anxiety (Lear and Pepper, 2015). This lack of clarity may be developmentally appropriate for university students who are navigating their sexual identity formation (Xu et al., 2021). Regarding the significant difference in sexual identity uncertainty between LG and Bi groups, it may stem from prevailing monosexist beliefs, which invalidate the authenticity of bisexuality and compel bisexual individuals to position themselves within a binary framework of sexual orientation (i.e., exclusively lesbian/gay or exclusively heterosexual) (Dyar et al., 2015; Chan and Leung, 2023). Likewise, individuals’ entitativity beliefs in the Chinese collectivistic culture may intensify their concerns about whether they align with specific group standards (Morandini et al., 2015), thereby increasing identity uncertainty. In this study, by highlighting the predictive role of identity centrality for uncertainty, we further clarify the interrelations among different dimensions of sexual identity in explaining group differences in psychological risk mechanisms between lesbian/gay and bisexual individuals.

### 4.2 Identity centrality as a core mechanism of risk

Sexual identity centrality, though not directly associated with mental health, was associated with depressive symptoms in two opposing ways: negatively through less sexual identity uncertainty, and positively through more experiences of interpersonal discrimination. Greater identity centrality may propel individuals to explore and/or express their sexual orientation more proactively, such as actively seeking relevant knowledge, coming out, and experiencing more conflict with societal expectations (Dyar et al., 2015; Griffith and Hebl, 2002; Hinton et al., 2022; Mohr and Kendra, 2011), thus simultaneously affirming their identity yet increasing the risk of experiencing interpersonal discrimination among bisexual individuals. These double-edged sword effects echo previous findings that identity centrality can promote better mental health via increased LGBTQ+ community connectedness, while heightening vulnerability to stigma sensitivity (Hinton et al., 2024).

Further research is needed to understand why bisexual individuals report lower sexual identity centrality than LG individuals. There are two possible explanations: (1) bisexual individuals may be at an earlier stage of sexual orientation identity development compared to lesbian and gay individuals, as a recent systematic review found that bisexual people experience identity milestones (e.g., attraction, self-identifying) about 1 or 2 years later than gay/lesbian people (Hall et al., 2021). (2) Despite growing societal acceptance towards LG people and increasing access to LGBTQ-related community resources, stigma and misunderstanding about bisexuality continue to persist (GLAAD Media Institute, 2024). Heightened social stigma may lead bisexual people to develop greater internalized binegativity (IB) and non-acceptance toward their personal and group identities. For instance, a recent study of Chinese bisexual individuals distinguished three IB-based identity profiles: group-dissonant, self-dissonant, and self-appreciating, indicating heterogeneity in how bisexual individuals negotiate personal affirmation and group belonging (Wei et al., 2025b). Thus, examining the potential association between identity centrality and internalized binegativity may further clarify identity-based risk processes.

### 4.3 Gender variation in risk pathways

Gender may influence psychological risk mechanisms in multiple ways within LG and Bi subgroups. Firstly, across the full LGB sample, women reported higher identity uncertainty but lower levels of interpersonal discrimination than men, both of which were associated with elevated psychological risk. The higher identity uncertainty among LGB women than men supports prior finding that exclusive same-sex attraction has been shown to be less stable among women than men (Diamond, 2016), which is partly due to women’s greater contextual sensitivity in sexual attraction and their tendency to anchor attraction in relational bonds rather than categorical gender distinctions. Moreover, the gender difference in identity uncertainty appears to be less pronounced in the bisexual group than in the LG group. This finding lends indirect support to earlier work suggesting that Chinese bisexual individuals of different genders exhibited no significant differences in sexual identity development milestones (Wei et al., 2025a). In terms of interpersonal discrimination experiences, gender disparity was observed only among gay men and lesbian women, not among bisexual individuals, which echoed Chan and Leung’s (2023) findings for Chinese bisexual people. It’s worth noting that gender did not significantly predict depressive symptoms in the hierarchical regression models, whereas its direct path to depressive symptoms became significant in the SEM after accounting for identity uncertainty, identity centrality, and interpersonal hostility (see Table 5). This pattern suggests that the association between gender and depressive symptoms may be partially obscured at the observed-score level and may become more evident once relevant mediating processes are modeled simultaneously.

Importantly, although gender-stratified analyses suggested that these paths from identity centrality to interpersonal discrimination and identity uncertainty were significant only among women, formal between-group comparisons indicated that the differences were not statistically significant. These findings suggest that the apparent significance in the female group is more likely attributable to sample size or statistical power, rather than reflecting true gender-specific effects, thereby supporting the robustness of these paths across gender. Alternatively, given that our sample consisted solely of highly educated Chinese young adults, these results may suggest that gender effects are less pronounced compared to other sociocultural influences.

### 4.4 Implications

The findings add to our understanding of the complexity of young Chinese sexual minority adults’ identity characteristics and experiences of interpersonal discrimination, highlighting differential mental health risk mechanisms among LGB university students, and inform intervention practices. Clinically, it is important to assess clients’ levels of identity centrality and uncertainty to better understand how interpersonal discrimination influences their depressive symptoms. For bisexual clients specifically, clinicians should assess discrimination experiences arising from both heterosexual and LG groups, as these multiple sources of bias may uniquely intensify risk. Notably, interpersonal discrimination showed the strongest effect on depressive symptoms, underscoring the need to prioritize coping with such discrimination as a critical intervention target for this population. Beyond clinical practice, the findings also point to the importance of reducing stigma against sexual minority students both within universities and in society more broadly, as stigma creates the social conditions that sustain identity uncertainty, interpersonal discrimination, and associated mental health risks.

### 4.5 Limitations

This study had several limitations, some of which suggest directions for future research. First, the non-proportional sampling method inevitably affected the results. The sample was drawn from university students in southwestern China, limiting the generalizability of the findings to other LGB populations. Regarding demographic information, we used education level as a proxy for age because the age distribution of university students in China is relatively concentrated; however, this limited our ability to assess the influence of age on the key variables. Additionally, combining sexual orientation and gender identity into a single question may conflate distinct constructs and limit the inclusion of non-binary individuals in this study. Finally, the cross-sectional design limits our ability to draw strong conclusions about causal direction. Although the proposed model is theoretically informed, alternative model specifications remain plausible, such as potential interaction effects between uncertainty and identity centrality.

To better capture the diversity of sexual identification, future research should consider identity developmental trajectories and apply person-centered approaches to identify distinct clusters of identity characteristics and minority stressors (Bergman and Magnusson, 1997) and their associations with mental health (Shramko et al., 2018; Wei et al., 2025b). Future studies should also investigate how societal stigma toward sexual and gender minorities shapes identity uncertainty and identity centrality, how levels of outness shape bisexual people’s experiences of interpersonal discrimination, and how these processes contribute to mental health disparities.

## 5 Conclusion

This study sheds light on how minority stress affects depressive symptoms differently among bisexual (Bi) and lesbian and gay (LG) Chinese university students. By examining the joint roles of sexual identity centrality, identity uncertainty, and interpersonal discrimination across groups, the findings demonstrate that the pathways linking identity-related processes to mental health are not uniform within sexual minority populations. Specifically, this study identified the central role of identity centrality in both intrapersonal and interpersonal stress processes associated with depression symptoms. These results highlight the importance of moving beyond treating LGB individuals as a monolithic group and the need for more identity-sensitive interventions, especially in cultural contexts where identity development and stigma may unfold differently. Overall, these findings show that accounting for identity characteristics is essential to understanding how minority stress influences mental health.

## Data Availability

The raw data supporting the conclusions of this article will be available by contacting the corresponding author.

## Footnotes

1 Although preliminary analyses suggested small differences in identity centrality and identity uncertainty across some major categories, major did not show stable effects in the regression analyses and was not significantly associated with sexual orientation or depressive symptoms. Therefore, it was not retained in the primary SEM model to preserve model parsimony.
