# Supplementary material for: Identity centrality as a double-edged sword: mental health mechanisms among lesbian/gay and bisexual university students in China
Source: Front Psychol. 2026 Jun 16;17:1737042. doi: 10.3389/fpsyg.2026.1737042 (PMC13316832; doi:10.3389/fpsyg.2026.1737042)
Supplement: Supplementary file 1 [file Supplementary_file_1.DOCX]

Table S1 Single-group CFA results for the Hostility subscale

| Group | Item | Standardized loading (λ) | p |
| --- | --- | --- | --- |
| Bi | I1 | .79 | < .001 |
|  | I2 | .91 | < .001 |
|  | I3 | .87 | < .001 |
| LG | I1 | .87 | < .001 |
|  | I2 | .88 | < .001 |
|  | I3 | .84 | < .001 |

As shown in Tables S1 the Hostility subscale demonstrated a clear unidimensional structure in both groups, with high factor loadings. The model is just-identified; therefore, model fit indices are not informative.

We then conducted a multi-group CFA to examine the invariance of the Hostility subscale across LG and Bi participants. Because the three-item one-factor model is just-identified at the configural level (df = 0), the configural model necessarily showed perfect fit, and its fit indices were not substantively informative. This model was therefore treated as the baseline model for subsequent invariance testing.

The metric invariance model showed excellent fit to the data, χ²(2) = 1.501, p = .472, CFI = 1.000, RMSEA = .000, and SRMR = .037. Constraining the factor loadings to equality across groups did not produce meaningful deterioration in model fit, supporting metric invariance.

The scalar invariance model also showed good overall fit, χ²(4) = 6.473, p = .167, CFI = .988, RMSEA = .058, and SRMR = .048. The change in approximate fit indices from the metric to scalar model was borderline, slightly exceeding the conventional cutoffs (ΔCFI = -.012, ΔSRMR = .011). The RMSEA also increased from .000 to .058 (ΔRMSEA = .058), which should be interpreted cautiously given the very small degrees of freedom in both models. Thus, scalar invariance is marginally supported: the scalar model showed a small decrease in CFI (ΔCFI = −.012) and an increase in SRMR (ΔSRMR = .011), both slightly exceeding conventional cutoff values; other indices (χ², RMSEA) indicate acceptable fit. Taken together, the findings provide strong support for metric invariance and tentative support for scalar invariance. Strict invariance was not tested, as it is not required for mean comparisons.

Table S2 One-way ANOVA Results for Differences in Key Variables by Major

| Variable | *F*(3, 359) | *p* |
| --- | --- | --- |
| Depressive symptoms | 0.08 | 0.973 |
| Identity uncertainty | 1.68 | 0.171 |
| Identity centrality | 2.86* | 0.037 |
| Interpersonal discrimination | 0.2 | 0.899 |

*Note:* ^*^*p* < .05, ^**^*p* < .01, ^***^*p* < .001.

One-way ANOVAs showed a significant group difference in identity centrality across major groups, *F*(3, 359) = 2.86, p = .037. Post hoc comparisons were therefore conducted for identity centrality only. Bonferroni-adjusted post hoc comparisons indicated that participants in Major Group 1 (Humanities) reported significantly higher identity centrality than those in Major Group 2 (Science and Engineering), mean difference = 0.405, SE = 0.144, p = .030, 95% CI [0.024, 0.787]. No other pairwise comparisons were significant.

Table S3 One-way ANOVA Results for Differences in Key Variables by Parents’ education level

| Variable | F(3, 359) | p |
| --- | --- | --- |
| Depressive symptoms | 2.42 | .066 |
| Identity uncertainty | 1.60 | .189 |
| Identity centrality | 2.16 | .093 |
| Interpersonal discrimination | 0.12 | .948 |

Parents’ education level was coded as a four-category grouping variable. Preliminary one-way ANOVAs showed no statistically significant differences across parental education groups in the key study variables, although depressive symptoms and identity centrality approached significance, *F*(3, 359) = 2.42, *p* = .066, and *F*(3, 359) = 2.16, *p* = .093, respectively.

Further, parental education was coded into a continuous variable, with higher scores indicating higher levels of parental educational attainment. This variable was significantly correlated with gender in the correlational analysis and was therefore included as a covariate in the subsequent hierarchical regression analyses.

### Table S4 One-way ANOVA Results for Differences in Key Variables by **Place of Origin**

| Variable | F(3, 359) | p |
| --- | --- | --- |
| Depressive symptoms | 1.69 | .185 |
| Identity uncertainty | 2.02 | .135 |
| Identity centrality | 0.19 | .828 |
| Interpersonal discrimination | 0.44 | .642 |

Preliminary one-way ANOVAs showed no significant differences across place-of-origin groups in depressive symptoms, identity uncertainty, identity centrality, or interpersonal discrimination, *F*s(3, 359) = 0.19–2.02, *p*s = .135–.828. Therefore, place of origin was not included as a covariate in subsequent regression analyses.

Table S5 **Independent-samples t Test Results by Education Level**

| Variable | t | df | p |
| --- | --- | --- | --- |
| Depressive symptoms | 0.60 | 361 | .548 |
| Identity uncertainty | -0.92 | 361 | .359 |
| Identity centrality | -0.13 | 361 | .897 |
| Interpersonal discrimination | 1.68 | 361 | .095 |

Preliminary independent-samples t tests showed no significant differences by education level in depressive symptoms, identity uncertainty, identity centrality, or interpersonal discrimination, ts(361) = -0.92–1.68, ps = .095–.897. This variable was also not significantly correlated with the key variables in the correlational analysis. Therefore, education level was not included as a covariate in subsequent regression analyses.

Table S6 Model fit indices for the gender-stratified structural equation models

| Group | n | χ² | df | CFI | TLI | RMSEA | SRMR |
| --- | --- | --- | --- | --- | --- | --- | --- |
| Women | 224 | 458.02 | 222 | .902 | .889 | .069 | .063 |
| Men | 139 | 343.49 | 222 | .926 | .916 | .063 | .067 |
| Multi-group | 363 | 891.286 | 486 | .885 | .880 | .068 | .089 |

*Note:* All models showed acceptable fit, with slightly better fit for the male group.

## Table S7 Direct structural paths in the gender-stratified SEMs (STDYX standardized estimates)

| Path | Men Estimate | S.E. | p | Women Estimate | S.E. | p |
| --- | --- | --- | --- | --- | --- | --- |
| Centra → Unce | -0.139 | 0.097 | .152 | -0.244* | 0.106 | .022 |
| Centra → IPH | 0.134 | 0.108 | .213 | 0.305** | 0.099 | .002 |
| Unce → Depre | 0.158 | 0.109 | .149 | 0.202* | 0.088 | .022 |
| Centra → Depre | -0.177 | 0.113 | .117 | 0.099 | 0.101 | .327 |
| IPH → Depre | 0.375** | 0.114 | .001 | 0.283** | 0.099 | .004 |
| SO → Unce | 0.400*** | 0.094 | < .001 | 0.156† | 0.090 | .083 |
| SO → Centra | -0.455*** | 0.082 | < .001 | -0.456*** | 0.069 | < .001 |
| SO → IPH | -0.332*** | 0.090 | < .001 | -0.095 | 0.093 | .310 |
| SO → Depre | -0.147 | 0.125 | .238 | 0.066 | 0.072 | .361 |

*Note.* SO = Sexual Orientation, IPH = Interpersonal discrimination, Centra=centrality, Unce=uncertainty, Depre = Depressive symptoms. ^*^*p* < .05, ^**^*p* < .01, ^***^*p* < .001.

Table S8 Indirect effects in the gender-stratified SEMs (unstandardized estimates with 95% bootstrap CIs)

| Indirect path | Men Estimate | 95% CI | Women Estimate | 95% CI |
| --- | --- | --- | --- | --- |
| SO → Unce → Depre (ind1) | 0.084 | [-0.030, 0.227] | 0.037 | [-0.005, 0.104] |
| SO → Centra → Depre (ind2) | 0.107 | [-0.029, 0.263] | -0.054 | [-0.177, 0.051] |
| SO → IPH → Depre (ind3) | -0.165* | [-0.321, -0.045] | -0.032 | [-0.107, 0.028] |
| SO → Centra → Unce → Depre (ind4) | 0.013 | [-0.008, 0.057] | 0.027* | [0.001, 0.072] |
| SO → Centra → IPH → Depre (ind5) | -0.030 | [-0.092, 0.019] | -0.047* | [-0.106, -0.007] |
| Centra → Unce → Depre (chain_Centra_Unce) | -0.028 | [-0.139, 0.017] | -0.028* | [-0.074, -0.001] |
| Centra → IPH → Depre (chain_Centra_IPH) | 0.063 | [-0.043, 0.323] | 0.049* | [0.008, 0.116] |
| Total indirect effect | 0.009 | [-0.237, 0.247] | -0.068 | [-0.190, 0.054] |
| Total effect | -0.187 | [-0.431, 0.047] | 0.010 | [-0.167, 0.171] |

*Note.* SO = Sexual Orientation, IPH = Interpersonal discrimination, Centra=centrality, Unce= uncertainty, Depre = Depressive symptoms. ^*^*p* < .05, ^**^*p* < .01, ^***^*p* < .001.
