# Supplementary material for: Identity centrality as a double-edged sword: mental health mechanisms among lesbian/gay and bisexual university students in China
Source: Front Psychol. 2026 Jun 16;17:1737042. doi: 10.3389/fpsyg.2026.1737042 (PMC13316832; doi:10.3389/fpsyg.2026.1737042)
Supplement: Supplementary file 2 [file Supplementary_file_2.DOCX]

Mplus Code

1. CFA of the Hostility subscale

TITLE: The unidimensional structure of the Hostility sub-scale separately in the Bi and LG groups

DATA: FILE=Bi.CSV; ! 可替换为DATA: FILE=LG.CSV;

VARIABLE: NAMES ARE ID Gen D1-D10 u1-u4 C1-C5 I1-I3;

USEVARIABLES ARE I1-I3; ! 本次分析中使用的变量

ANALYSIS:

BOOTSTRAP = 5000; ! 用bootstrap做间接效应置信区间

ESTIMATOR = ML; ! 最大似然估计

MODEL:

IPH BY I1-I3;

OUTPUT:

CINTERVAL(BOOTSTRAP);

STDYX TECH4; ! 输出标准化系数

TITLE: Configural invariance of Hostility across LG and BI;

VARIABLE:

NAMES ARE ID SO Gen D1-D10 u1-u4 C1-C5 I1-I3 ;

USEVARIABLES = I1-I3;

GROUPING = SO (0 = LG 1 = Bi);

ANALYSIS: ESTIMATOR = MLR;

MODEL:

  IPH BY I1* I2 I3;

  IPH@1; ! 固定潜变量方差为1

  [IPH@0]; ! 固定潜变量均值为0

MODEL Bi:

  IPH BY I1* I2 I3;

  IPH@1;

  [IPH@0];

  [I1-I3];

OUTPUT: STDYX;

TITLE: Metric of Hostility across LG and BI;

DATA: FILE = LGB.CSV;

VARIABLE:

NAMES ARE ID SO Gen D1-D10 u1-u4 C1-C5 I1-I3;

USEVARIABLES = SO I1-I3;

GROUPING = SO (0 = LG 1 = Bi);

ANALYSIS:

ESTIMATOR = MLR;

MODEL:

IPH BY I1* I2 I3;

IPH@1; ! 第一组固定方差

[IPH@0]; ! 第一组固定均值

MODEL Bi:

IPH*; ! 第二组方差自由

[IPH@0]; ! 第二组均值固定

[I1-I3]; ! 截距在组间保持自由

OUTPUT:

STANDARDIZED MODINDICES(10);

TITLE: Scalar Invariance of Hostility across LG and BI;

DATA: FILE = LGB.CSV;

VARIABLE:

NAMES ARE ID SO Gen D1-D10 u1-u4 C1-C5 I1-I3;

USEVARIABLES = I1-I3;

GROUPING = SO (0 = LG 1 = Bi);

ANALYSIS:

ESTIMATOR = MLR;

MODEL:

IPH BY I1* I2 I3;

IPH@1; ! 参照组方差固定为 1

[IPH@0]; ! 参照组均值固定为 0

MODEL Bi:

IPH;

[IPH];

OUTPUT:

sampstat standardized residual modindices (3.84);

1. Confirmatory factor analysis (CFA) model for multiple meditation model

DATA: FILE=LGB.CSV;

VARIABLE: NAMES ARE ID SO Gen D1-D10 u1-u4 C1-C5 I1-I3;

USEVARIABLES ARE D1-D10 u1-u4 C1-C5 I1-I3; !

ANALYSIS:

TYPE = GENERAL; ! 修改为SEM以获得拟合指标

ESTIMATOR = ML; ! 最大似然估计

MODEL:

Depre BY D1-D10;

Unce BY u1-u4;

Centra BY C1-C5;

IPH BY I1-I3;

OUTPUT: STDYX TECH4; ! 输出标准化结果和模型拟合

1. Multiple mediation model controlling for gender

DATA: FILE=LGB.CSV;

VARIABLE: NAMES ARE ID SO Gen D1-D10 u1-u4 C1-C5 I1-I3;

USEVARIABLES ARE SO Gen D1-D10 u1-u4 C1-C5 I1-I3; ! 本次分析中使用的变量

ANALYSIS:

BOOTSTRAP = 5000; ! 用bootstrap做间接效应置信区间

ESTIMATOR = ML; ! 最大似然估计

MODEL:

Depre BY D1-D10;

Unce BY u1-u4;

Centra BY C1-C5;

IPH BY I1-I3;

! 中介间的链式路径

Unce ON SO (a1)

Centra (a4)

Gen;

Centra ON SO (a2)

Gen;

IPH ON SO (a3)

Centra (a5)

Gen;

Depre ON SO (c_prime)

Unce (b1)

Centra (b2)

IPH (b3)

Gen;

IPH WITH Unce;

MODEL INDIRECT:

Depre IND SO; ! 自动输出所有可用的间接效应

MODEL CONSTRAINT:

NEW(ind1 ind2 ind3 ind4 ind5

chain_Centra_Unce chain_Centra_IPH

total_indirect total_effect);

! 单独链：SO → Unce → DPRE

ind1 = a1 * b1;

! 单独链：SO → Centra → DPRE

ind2 = a2 * b2;

! 单独链：SO → IPH → DPRE

ind3 = a3 * b3;

! 链式路径：SO → Centra → Unce → DPRE

ind4 = a2 * a4 * b1;

! 链式路径：SO → Centra → IPH → DPRE

ind5 = a2 * a5 * b3;

! 特别关注 Centra 本身的次级链式效应

chain_Centra_Unce = a4 * b1;

chain_Centra_IPH = a5 * b3;

! 总间接效应 = 所有路径之和

total_indirect = ind1 + ind2 + ind3 + ind4 + ind5;

! 总效应 = 总间接效应 + 直接效应

total_effect = total_indirect + c_prime;

OUTPUT:

CINTERVAL(BOOTSTRAP);

STDYX TECH4; ! 输出标准化系数

1. Gender-stratified SEM

DATA: FILE=LGB-M.CSV; ! 可替换为DATA: FILE=LGB-F.CSV;

VARIABLE: NAMES ARE ID SO D1-D10 u1-u4 C1-C5 I1-I3;

USEVARIABLES ARE SO D1-D10 u1-u4 C1-C5 I1-I3; ! 本次分析中使用的变量

ANALYSIS:

BOOTSTRAP = 5000; ! 用bootstrap做间接效应置信区间

ESTIMATOR = ML; ! 最大似然估计

MODEL:

Depre BY D1-D10;

Unce BY u1-u4;

Centra BY C1-C5;

IPH BY I1-I3;

Unce ON SO (a1);

Centra ON SO (a2);

IPH ON SO (a3);

! 中介间的链式路径

Unce ON Centra (a4);

IPH ON Centra (a5);

! 中介对 Depression

Depre ON Unce (b1);

Depre ON Centra (b2);

Depre ON IPH (b3);

Depre ON SO (c_prime);

MODEL INDIRECT:

Depre IND SO; ! 自动输出所有可用的间接效应

MODEL CONSTRAINT:

NEW(ind1 ind2 ind3 ind4 ind5

chain_Centra_Unce chain_Centra_IPH

total_indirect total_effect);

! 单独链：SO → Unce → DPRE

ind1 = a1 * b1;

! 单独链：SO → Centra → DPRE

ind2 = a2 * b2;

! 单独链：SO → IPH → DPRE

ind3 = a3 * b3;

! 链式路径：SO → Centra → Unce → DPRE

ind4 = a2 * a4 * b1;

! 链式路径：SO → Centra → IPH → DPRE

ind5 = a2 * a5 * b3;

! 特别关注 Centra 本身的次级链式效应

chain_Centra_Unce = a4 * b1;

chain_Centra_IPH = a5 * b3;

! 总间接效应 = 所有路径之和

total_indirect = ind1 + ind2 + ind3 + ind4 + ind5;

! 总效应 = 总间接效应 + 直接效应

total_effect = total_indirect + c_prime;

OUTPUT:

CINTERVAL(BOOTSTRAP);

STDYX TECH4; ! 输出标准化系数

1. Multi-group Comparisons by Gender

TITLE: Multi-group SEM by gender:

Testing gender differences in the paths;

DATA:

FILE = LGB.CSV;

VARIABLE:

NAMES = ID Gen SO D1-D10 u1-u4 C1-C5 I1-I3;

USEVARIABLES = SO Gen D1-D10 u1-u4 C1-C5 I1-I3;

GROUPING = Gen (0 = men 1 = women);

ANALYSIS:

ESTIMATOR = MLR;

MODEL:

Depre BY D1-D10;

Unce BY u1-u4;

Centra BY C1-C5;

IPH BY I1-I3;

Unce ON SO Centra;

IPH ON SO Centra;

Depre ON Unce;

MODEL men:

Unce ON Centra (a1);

IPH ON Centra (b1);

Unce ON SO (c1);

IPH ON SO (d1);

Depre ON Unce (e1);

MODEL women:

Unce ON Centra (a2);

IPH ON Centra (b2);

Unce ON SO (c2);

IPH ON SO (d2);

Depre ON Unce (e2);

MODEL CONSTRAINT:

NEW(diff_Unce diff_IPH diff_SO1 diff_SO2 diff_Depre);

diff_Unce = a1 - a2;

diff_IPH = b1 - b2;

diff_SO1 = c1 - c2;

diff_SO2 = d1 - d2;

diff_Depre = e1-e2;

OUTPUT:

STANDARDIZED

TECH1

TECH4;
