# Supplementary material for: Identity centrality as a double-edged sword: mental health mechanisms among lesbian/gay and bisexual university students in China
Source: Front Psychol. 2026 Jun 16;17:1737042. doi: 10.3389/fpsyg.2026.1737042 (PMC13316832; doi:10.3389/fpsyg.2026.1737042)
Supplement: Supplementary file 3 [file Table_1.DOCX]

**Identity Centrality as a Double-Edged Sword: Mental Health Mechanisms Among Lesbian/Gay and Bisexual University Students in China**

Shanshan Zhang^12^ Xintong Yao^3^ Kyle Tan^4^ Chongzheng Wei^5^

^1^Marxism School, Sichuan University, Chengdu 610065, China

^2^ College of Education for the Future, Beijing Normal University, Zhuhai 519087, China

^3^ School of Psychological and Social Sciences, University of Waikato, Hamilton 3240, New Zealand

^4^ Faculty of Māori and Indigenous Studies, University of Waikato, Hamilton 3240, New Zealand

^5^ Department of Counseling Psychology, Santa Clara University, California, USA 95050

# **Author Note**

This study was supported by the Higher Education Teaching Reform Project (Phase 10) of Sichuan University (Project No. SCU10239).

The authors have no conflicts of interest to disclose.
