# Supplementary material for: Identity centrality as a double-edged sword: mental health mechanisms among lesbian/gay and bisexual university students in China
Source: Front Psychol. 2026 Jun 16;17:1737042. doi: 10.3389/fpsyg.2026.1737042 (PMC13316832; doi:10.3389/fpsyg.2026.1737042)
Supplement: Supplementary file 4 [file Table_2.DOCX]

**›Table 1** *Demographics of the Participants*

|  | **Total (n = 363)** | **Bi (n = 221)** | **LG (n = 142)** | ***Group***  ***difference χ²/t-value*** |
| --- | --- | --- | --- | --- |
|  | **N (%)/**  **Mean (S.D.)** | **N (%)/**  **Mean (S.D.)** | **N (%)/**  **Mean (S.D.)** |  |
| Gender, *n*(%) |  |  |  | 73.03^***^ |
| Women | 224 (61.71) | 175 (79.19) | 49 (34.51) |  |
| Men | 139 (38.29) | 46 (20.81) | 93 (65.49) |  |
| Education level |  |  |  | 0.13 |
| Postgraduates | 75 (20.66) | 47 (21.27) | 28 (19.72) |  |
| Undergraduates | 288 (79.34) | 174 (78.73) | 114 (80.28) |  |
| Major |  |  |  | 3.45 |
| Humanities | 118 (32.5%) | 70 (31.67%) | 48 (33.8%) |  |
| Science and Engineering | 157 (43.3%) | 91 (41.18%) | 66 (46.48%) |  |
| Medicine | 60 (16.5%) | 39 (17.65%) | 21 (14.79%) |  |
| Arts and Sports | 28 (7.7%) | 21 (9.5%) | 7 (4.93%) |  |
| Place of origin |  |  |  | 0.44 |
| Rural | 86 (23.7%) | 51 (23.08%) | 35 (24.65%) |  |
| Township | 80 (22%) | 47 (21.27%) | 33 (23.24%) |  |
| Urban | 197 (54.3%) | 123 (55.66%) | 74 (52.11%) |  |
| Parents’ education level |  |  |  | 5.35 |
| Both under High school education | 72 (19.8%) | 42 (19%) | 30 (21.13%) |  |
| One has High school education | 89 (24.5%) | 47 (21.27%) | 42 (29.58%) |  |
| One has College diploma or above | 60 (16.5%) | 36 (16.29%) | 24 (16.9%) |  |
| Both have College diploma or above | 142 (39.1%) | 96 (43.44%) | 46 (32.39%) |  |
| Centrality (1-6) | 3.21 ± 1.19 | 2.76 ± 1.03 | 3.89 ± 1.09 | 9.93^***^ |
| Uncertainty (1-6) | 2.51 ± 1.33 | 2.92 ± 1.28 | 1.87 ± 1.14 | -8.20^***^ |
| Interpersonal discrimination (1-6) | 1.72 ± 0.87 | 1.44 ± 0.66 | 2.15 ± 0.98 | 7.53^***^ |
| Depressive symptoms (1-4) | 2.10 ± 0.50 | 2.08 ± 0.50 | 2.13 ± 0.51 | 0.84 |

*Note:* LG = Lesbian Women and Gay men, Bi = Bisexual individuals.

^*^*p* < .05, ^**^*p* < .01, ^***^*p* < .001.

**Table 2** *Gender Differences in Key Study Variables*

| **Variable** | **Total** | | | | **LG** | | | | **Bi** | | | |
| --- | --- | --- | --- | --- | --- | --- | --- | --- | --- | --- | --- | --- |
|  | **Men M ± SD** | **Women M ± SD** | ***t*(df)** | ***p*** | **Men M ± SD** | **Women M ± SD** | ***t*(df)** | ***p*** | **Men M ± SD** | **Women M ± SD** | ***t*(df)** | ***p*** |
| Depressive symptoms | 2.08 ± 0.54 | 2.11 ± 0.48 | -0.53 (361) | 0.595 | 2.14 ± 0.52 | 2.11 ± 0.50 | 0.35 (140) | 0.73 | 1.97 ± 0.55 | 2.11 ± 0.48 | -1.76 (219) | 0.081 |
| Identity uncertainty | 2.10 ± 1.21 | 2.77 ± 1.34 | -4.81 (361) | **< .001** | 1.73 ± 1.02 | 2.14 ± 1.32 | -2.06 (140) | **0.041** | 2.85 ± 1.24 | 2.94 ± 1.29 | -0.46 (219) | 0.65 |
| Identity centrality | 3.50 ± 1.14 | 3.03 ± 1.18 | 3.74 (361) | **< .001** | 3.85 ± 1.04 | 3.98 ± 1.19 | -0.70 (140) | 0.487 | 2.79 ± 1.02 | 2.76 ± 1.04 | 0.20 (219) | 0.845 |
| Interpersonal discrimination | 2.06 ± 1.01 | 1.50 ± 0.69 | 5.74 (218.13) | **< .001** | 2.33 ± 1.01 | 1.80 ± 0.81 | 3.20 (140) | **0.002** | 1.52 ± 0.77 | 1.42 ± 0.63 | 0.91 (219) | 0.367 |

*Note:* significant *p* values are bolded.

**Table 3** *Intercorrelations among Variables*

| *Variables* | **1** | **2** | **3** | **4** | **5** | **6** | **7** | **α** |
| --- | --- | --- | --- | --- | --- | --- | --- | --- |
| 1. Uncertainty | — |  |  |  |  |  |  | 0.91 |
| 2. Centrality | -.318** | — |  |  |  |  |  | 0.8 |
| 3.Interpersonal discrimination | -.153** | .305** | — |  |  |  |  | 0.91 |
| 4.Depressive symptoms | .129* | 0.042 | .261** | — |  |  |  | 0.87 |
| 5. Gender | .245** | -.193** | -.313** | 0.028 | — |  |  |  |
| 1. Sexual orientation | .388** | -.463** | -.395** | -0.044 | .449** | — |  |  |
| 1. Education level | 0.048 | 0.007 | -0.088 | -0.032 | 0.052 | 0.019 | — |  |
| 1. Parents’ education level | -0.046 | -0.059 | -0.016 | -0.041 | .127* | 0.099 | -.182** |  |

*Note.* Binary variables were coded as follows: Sexual orientation (LG = 0, Bi = 1), Gender (men = 0, women = 1), and Education level (undergraduate = 0, postgraduate = 1). Parents’ education level was recoded into a four-point ordered variable and treated as a continuous variable in the analyses, with higher scores indicating higher levels of parental educational attainment. Because Major and Place of origin were multicategory categorical variables, their associations with the focal study variables were examined using one-way ANOVAs and the results were reported in the Supplementary Tables.

^*^*p* < .05, ^**^*p* < .01.

**Table 4** *Hierarchical Multiple* *Regression Model Results*

|  |  | **Depressive symptoms** | | **Uncertainty** | | **Centrality** | | **Interpersonal discrimination** | |
| --- | --- | --- | --- | --- | --- | --- | --- | --- | --- |
|  |  | **B (SE)** | ***β*** | **B (SE)** | ***β*** | **B (SE)** | ***β*** | **B (SE)** | ***β*** |
| **Block 1: Control Variables** | |  |  |  |  |  |  |  |  |
| Parents’ education level | | -0.14 (0.23) | -0.032 | -0.086 (0.056) | -0.076 | -0.010 (0.048) | -0.01 | 0.024 (0.036) | 0.032 |
| Major (Science and Engineering) | | -0.03 (0.61) | -0.003 | 0.228 (0.150) | 0.085 | -0.444 (0.130) | -0.185** | 0.045 (0.098) | 0.026 |
| Major (Medicine) | | -0.14 (0.78) | -0.01 | -0.077 (0.194) | -0.021 | -0.312 (0.168) | -0.098 | 0.107 (0.127) | 0.046 |
| Major (Arts and Sports) | | -0.21 (1.05) | -0.011 | -0.599 (0.260) | -0.12* | -0.050 (0.225) | -0.011 | 0.218 (0.169) | 0.067 |
| **Block 2:** | |  |  |  |  |  |  |  |  |
| **Sexual orientation** | |  |  |  |  |  |  |  |  |
| Bisexual (lesbian/Gay as reference) | | -0.89 (0.97) | -0.086 | 1.142 (0.220) | 0.42*** | -0.963 (0.191) | -0.396*** | -0.837 (0.144) | -0.471*** |
| **Gender** |  |  |  |  |  |  |  |  |  |
| Women (Men as reference) | | 0.44 (0.90) | 0.042 | 0.548 (0.216) | 0.201* | 0.109 (0.187) | 0.045 | -0.569 (0.141) | -0.319*** |
| **Interaction** | |  |  |  |  |  |  |  |  |
| (Sexual orientation x Gender) | | 1.13 (1.20) | 0.112 | -0.339 (0.295) | -0.128 | -0.281 (0.256) | -0.118 | 0.471 (0.192) | 0.271* |
| **Block 3:** |  |  |  |  |  |  |  |  |  |
| Uncertainty | | 0.62 (0.22) | 0.163** |  |  |  |  |  |  |
| Centrality | | 0.04 (0.25) | 0.01 |  |  |  |  |  |  |
| Interpersonal discrimination | | 1.73 (0.33) | 0.299*** |  |  |  |  |  |  |

*Note.* Major was dummy coded, with Humanities as the reference group. Parents’ education level was recoded into a four-point ordered variable and treated as a continuous variable in the analyses, with higher scores indicating higher levels of parental educational attainment.

^*^*p* < .05, ^**^*p* < .01, ^***^*p* < .001.

**Table 5**

*Parameter Estimates for the Hypothesized Model (Controlling Gender)*

| **Path** | ***Β/* Estimate** | **S.E.** | ***P* / 95% bootstrap CI** |
| --- | --- | --- | --- |
| **Direct path** | ***β*** | **S.E.** | ***p*** |
| Centrality → Uncertainity | -0.195** | 0.074 | 0.008 |
| Centrality → IPH | 0.194** | 0.073 | 0.008 |
| Uncertainity → Depressive symptoms | 0.178** | 0.068 | 0.009 |
| Centrality → Depressive symptoms | -0.018 | 0.075 | 0.814 |
| IPH → Depressive symptoms | 0.353*** | 0.076 | < .001 |
| SO → Uncertainity | 0.272*** | 0.071 | < .001 |
| SO → Centrality | -0.497*** | 0.056 | < .001 |
| SO → IPH | -0.236** | 0.069 | 0.001 |
| IPH ↔ Uncertainity | 0.042 | 0.076 | 0.583 |
| SO → Depressive symptoms | -0.027 | 0.072 | 0.705 |
| Gender → Uncertainity | 0.108† | 0.055 | 0.05 |
| Gender → IPH | -0.187** | 0.056 | 0.001 |
| Gender → Centrality | 0.045 | 0.06 | 0.454 |
| Gender → Depressive symptoms | 0.118* | 0.06 | 0.048 |
| **Indirect path** | **Estimate** | **S.E.** | **95% bootstrap CI** |
| SO → Uncertainty → Depressive symptoms | 0.054* | 0.026 | [0.013, 0.116] |
| SO → Centrality→ Depressive symptoms | 0.01 | 0.042 | [-0.073, 0.094] |
| SO → IPH → Depressive symptoms | -0.093** | 0.035 | [-0.174, -0.033] |
| Centrality → Uncertainty → Depressive symptoms | -0.023* | 0.013 | [-0.055, -0.003] |
| Centrality → IPH → Depressive symptoms | 0.046* | 0.022 | [0.011, 0.096] |
| SO→ Centrality → Uncertainty → Depressive symptoms | 0.019* | 0.011 | [0.002, 0.046] |
| SO→ Centrality → IPH → Depressive symptoms | -0.038* | 0.017 | [-0.078, -0.010] |
| Total indirect effect | -0.048 | 0.054 | [-0.152, 0.063] |
| Total effect | -0.079 | 0.071 | [-0.216, 0.057] |

*Note.* SO = Sexual Orientation, IPH = Interpersonal discrimination. β values are STDYX standardized coefficients for direct structural paths. Indirect effects are reported as unstandardized estimates with bias-corrected bootstrap 95% confidence intervals (5,000 resamples).

^*^*p* < .05, ^**^*p* < .01, ^***^*p* < .001.

Table 6 Between-group Comparisons of Key Structural Paths across Gender

| Path | Men Estimate  (β) | S.E. | *p* | Women Estimate  (β) | S.E. | *p* | Difference  (Men − Women) | S.E. | *p* |
| --- | --- | --- | --- | --- | --- | --- | --- | --- | --- |
| SO → Uncertainty | 1.094*** | 0.239 | 0 | 0.61* | 0.279 | 0.029 | 0.484 | 0.366 | .186 |
| SO → IPH | -0.484** | 0.148 | 0.001 | -0.187 | 0.121 | 0.121 | -0.297 | 0.183 | .106 |
| Centrality → Uncertainty | -0.205 | 0.153 | 0.18 | -0.414* | 0.192 | 0.031 | 0.209 | 0.239 | .382 |
| Centrality → IPH | 0.181† | 0.1 | 0.069 | 0.167* | 0.068 | 0.014 | 0.014 | 0.120 | .907 |
| Uncertainty → Depressive symptoms | 0.035 | 0.037 | 0.336 | 0.056† | 0.029 | 0.05 | -0.021 | 0.046 | .651 |

*Note.* SO = Sexual Orientation, IPH = Interpersonal discrimination. ^*^*p* < .05, ^**^*p* < .01, ^***^*p* < .001.

Figure 1  *Multiple Mediation SEM Diagram Controlling for Gender*


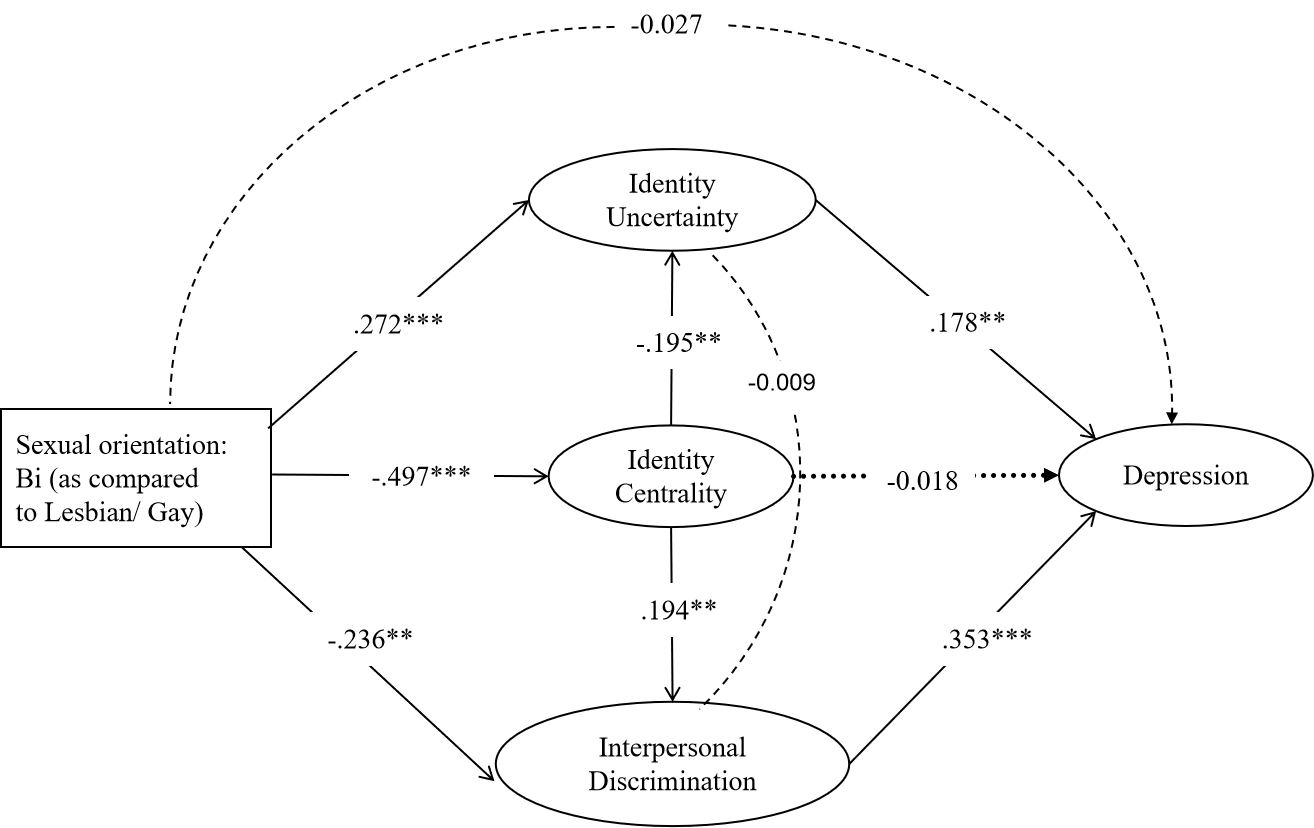


*Note.* ^*^*p* < .05, ^**^*p* < .01, ^***^*p* < .001.
